# Supplementary material for: CD137 (4-1BB) costimulation of CD8+ T cells is more potent when provided in cis than in trans with respect to CD3-TCR stimulation
Source: Nat Commun. 2021 Dec 15;12:7296. doi: 10.1038/s41467-021-27613-w (PMC8674279; doi:10.1038/s41467-021-27613-w)
Supplement: Supplementary file 1 — Supplementary Information [file 41467_2021_27613_MOESM1_ESM.pdf]

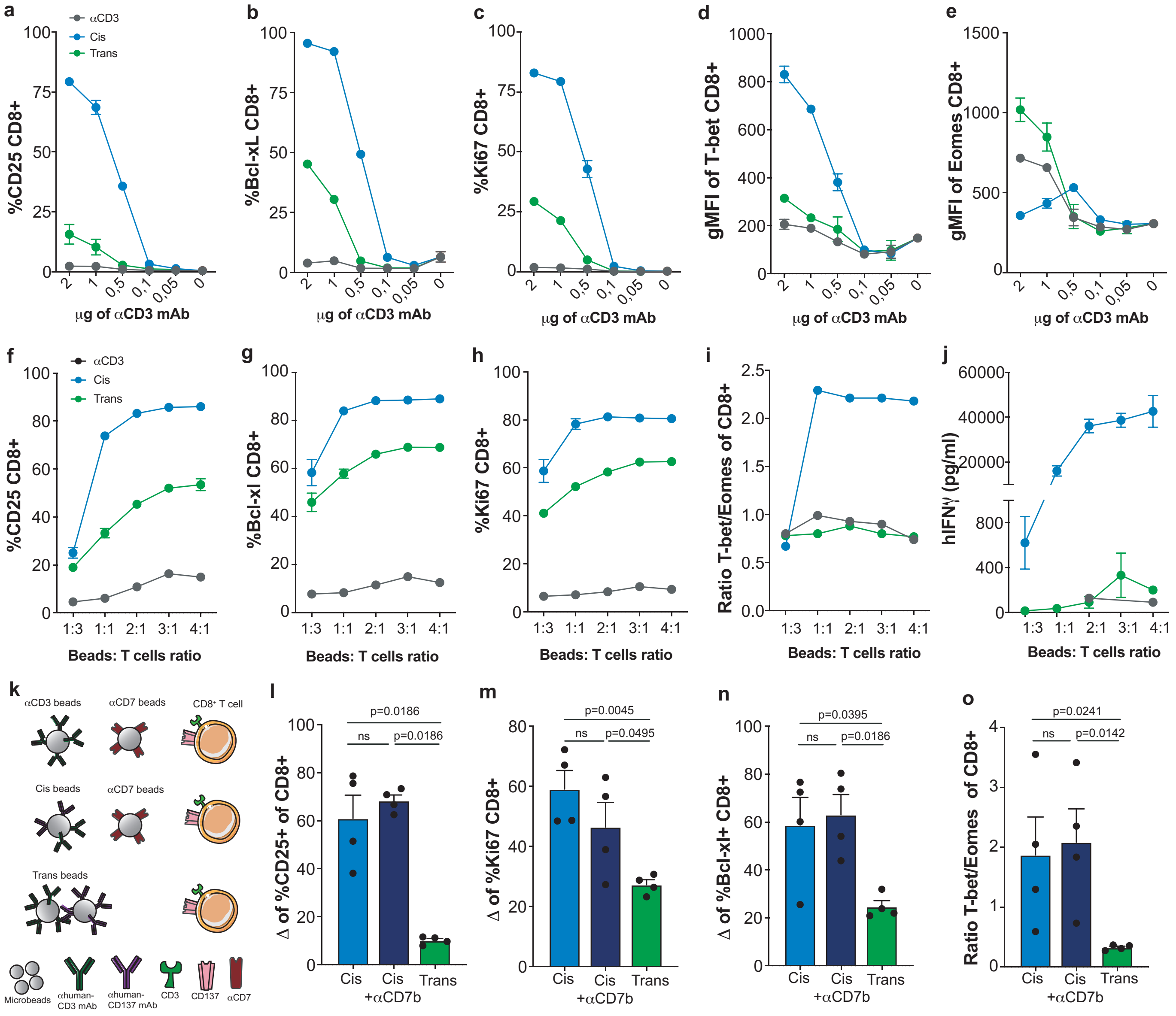


**Supplementary Figure 1**. **Cis and trans CD137-costimulation with different coating conditions of the microbeads**. Human primary CD8$^+$ T cells from healthy donors were activated with decreasing concentrations of αCD3 monoclonal antibody (mAb) used to coat the microbeads for 96 hours. Anti-CD137 mAb concentration exposed to microbeads was constant. Expression of cell-surface CD25 (**a**), intracellular Bcl-xL (**b**) and Ki67 (**c**) was analysed by flow cytometry in the indicated conditions of cis and trans costimulation. Flow cytometry measurement of T-bet (**d**) and Eomes (**e**) stainings are given as geometric mean fluorescence in FACS-gated CD8$^+$ T cells after cis versus trans costimulation. Data are representative of 6 independent donors. Human primary CD8$^+$ T cells from healthy donors were activated with different bead:T-cell ratios. Expression of cell-surface CD25 (**f**), intracellular Bcl-xL (**g**) and Ki67 (**h**) was determined by flow cytometry in the indicated conditions of cis and trans costimulation. **i**, summary data of the T-bet:Eomes ratio of CD8$^+$ T lymphocytes that was analysed by

flow cytometry. **j**, concentrations of IFNγ in the culture supernatants of the indicated experimental conditions. **k**, schematic layout of microbeads coated with anti-CD7 mAb used to stimulate $CD8^+$ T cells. Human primary $CD8^+$ T cells from healthy donors were activated with microbeads for 96 hours. Cell-surface CD25 (**l**) and intracellular (**m**) Bcl-xL and Ki67 (**n**) expression were analysed by flow cytometry in the indicated conditions (n=4). **o**, summary data showing the T-bet:Eomes ratio of $CD8^+$ T cells in cis versus trans costimulation (n=4). Data are representative of 4 independent donors. Summary data are given as the difference of the value between each costimulation condition subtracted from the anti-CD3 single stimulation condition in each case, showing that CD7 coated beads did not alter the effects of CD137 costimulation in cis. Data are given as mean ± s.e.m. Statistical significance was determined with the Friedman test with Dunn's correction in g to j (two-sided).

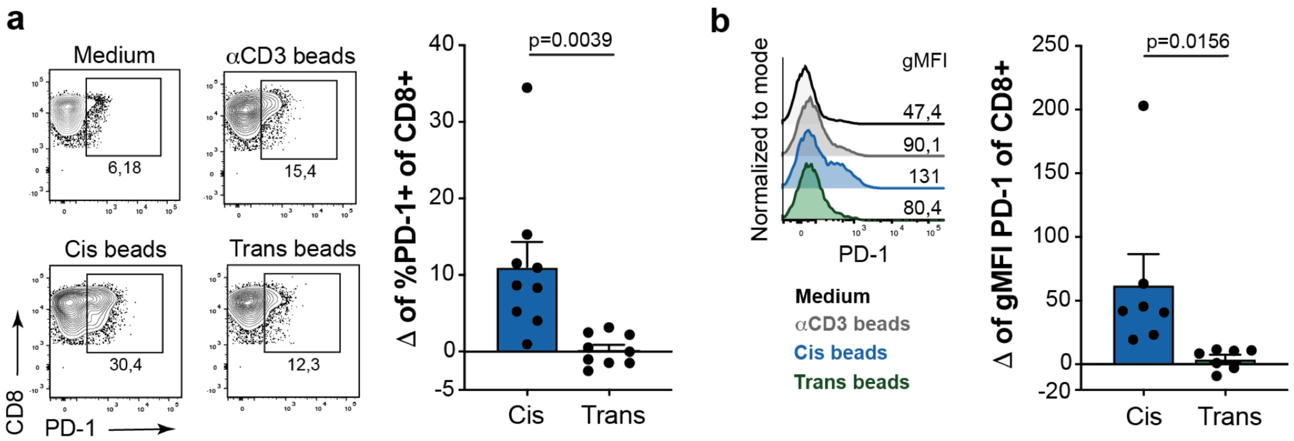


**Supplementary Figure 2. PD-1 expression on human CD8$^+$ T cells after CD137-costimulation in cis versus trans provided by antibody-coated microbeads** Representative flow cytometry dot plots (**a**) (n=8) and histograms (**b**) (n=7) showing PD-1 expression on primary human CD8$^+$ T cells activated with mAb coated beads. Numbers in the histograms indicate the geometric mean fluorescence intensity (gMFI). Summary data are given as mean ± s.e.m. Statistical significance was determined with paired t-test (two-sided).

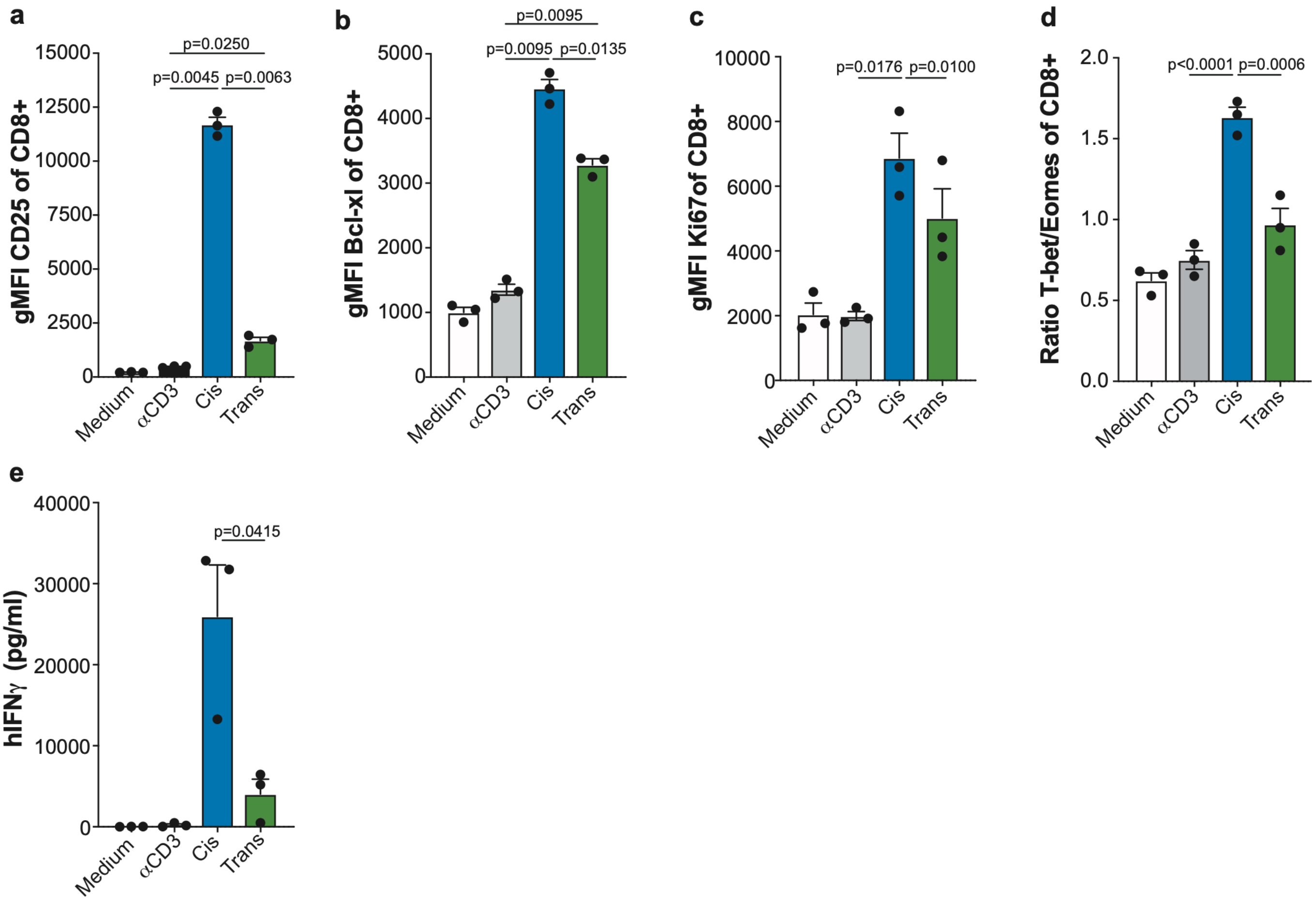


**Supplementary Figure 3**. **4-1BB Cis-costimulation outperforms trans CD137-costimulation also in T cells primed under CD28 costimulation.** Human primary $CD8^+$ T cells from healthy donors were pre-activated with plate-bound anti-CD3 and soluble anti-CD28 mAb for 16h and then rested without exogenous stimuli for 24h. Then T cells were stimulated for 72h with mAb coated microbeads providing 4-1BB costimulation either in cis or in trans. Flow cytometry measurement of CD25 (**a**), Bcl-xl (**b**), Ki67 (**c**) (n=3), stainings are given as geometric mean fluorescence intensity in FACS-gated $CD8^+$ T cells following cis or trans costimulation as indicated. **d**, summary data showing the T-bet:Eomes ratio of $CD8^+$ T cells in cis versus trans costimulation (n=3). **e**, concentrations of IFNγ in the culture supernatants of the indicated experimental conditions (n=3). Summary data are given as mean ± s.e.m. Statistical significance was determined with one-way Anova with Dunn's multiple comparison test (one-sided).

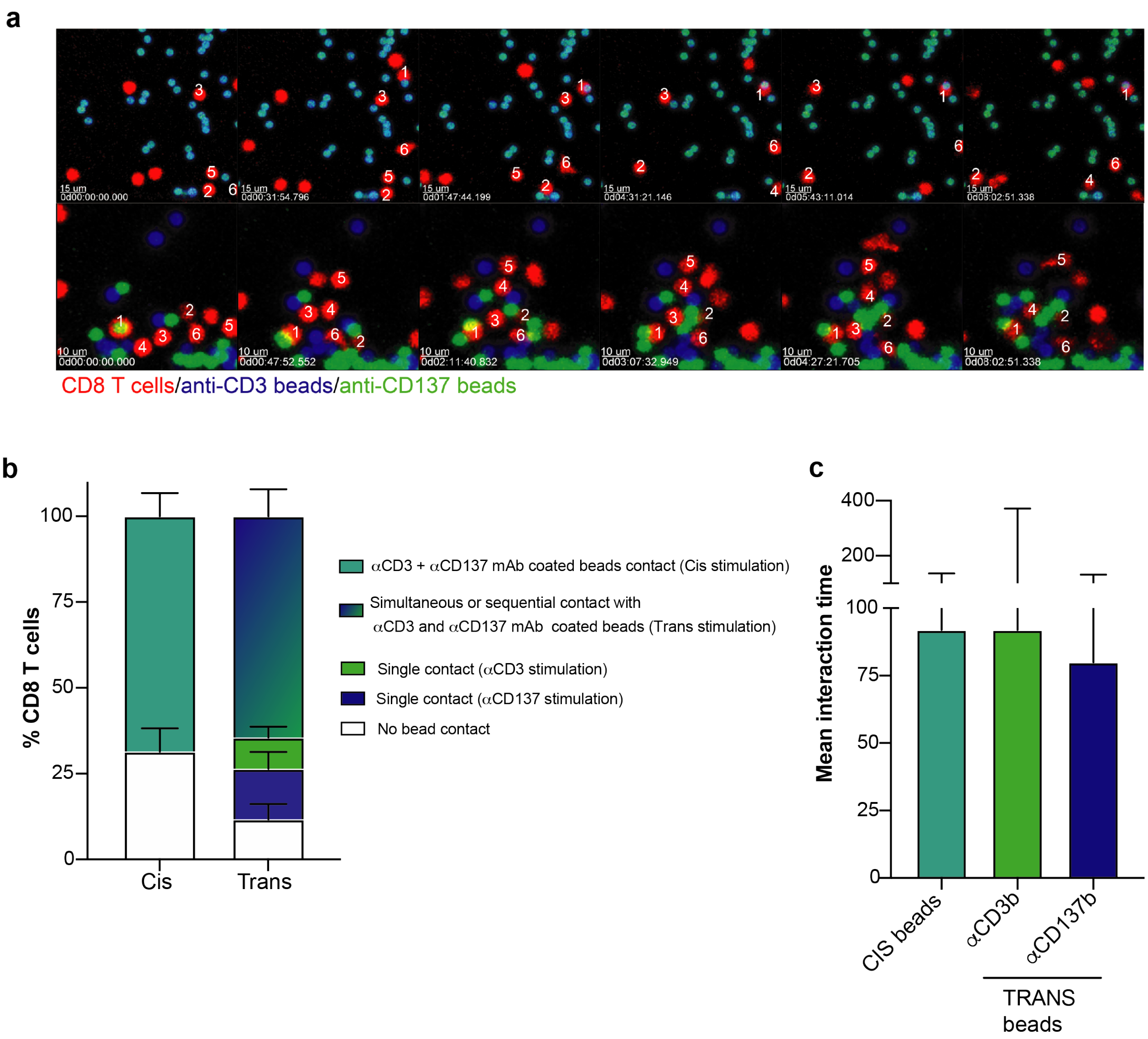


**Supplementary Figure 4. Efficient bead to T-cell interactions with mAb coated beads. a**, representative frames from videos of CD8$^+$ T cells (RED) co-cultured with mAb coated beads for 8 hours. Microbeads coated with αCD3-AF647 mAb are shown in blue, microbeads coated with αCD137-AF488 are shown in green, and microbeads coated with both αCD3-AF647 and αCD137-AF488 mAbs are shown in turquoise. Each digit in white indicates a single CD8$^+$ T cell (scale 15 µm). **b**, CD8$^+$ T cells were followed over time and tracks were used to quantify the percentage of cells interacting with microbeads coupled to both αCD3 and αCD137 mAbs (cis-stimulation), and percentage of cells interacting simultaneously or sequentially with both αCD3 and αCD137 mAb coated microbeads (trans-stimulation) (scale 10 µm).. **c**, summary of the T-cell:bead contact duration during CD8$^+$ T cell stimulation with cis and trans beads as observed in the time-lapse videos. Data are representative of two independent experiments. Data are given as mean ± s.e.m.

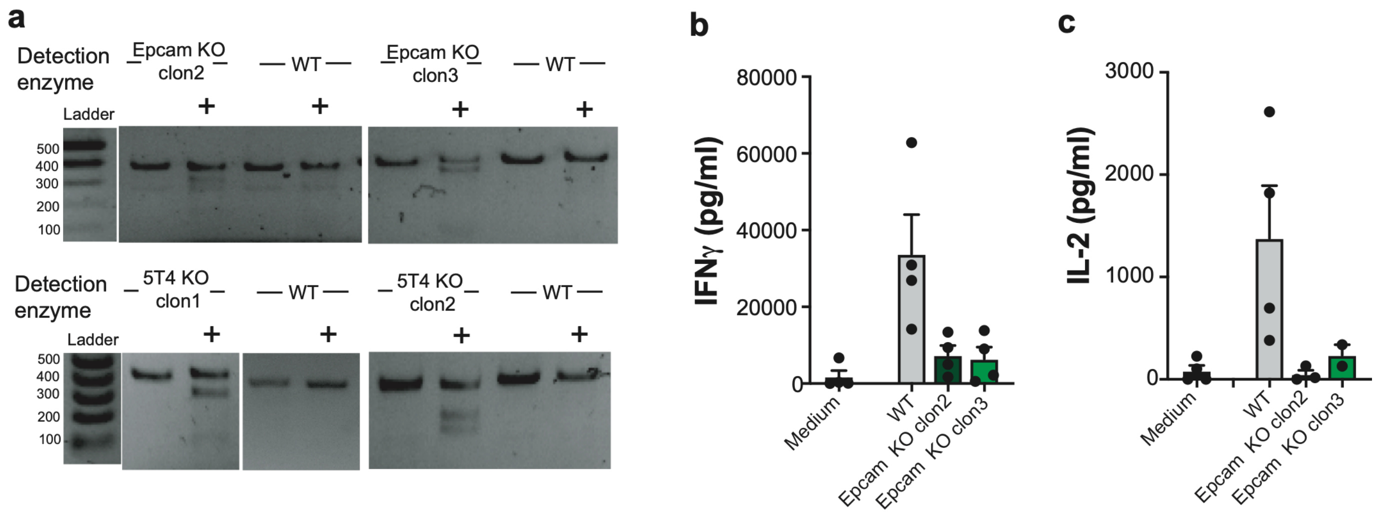


**Supplementary Figure 5. Generation of CRISPR/Cas9 silenced EpCAM and 5T4 HCT116 cells. a,** in vitro cleavage assay of EpCAM and 5T4 loci. **b**, $CD8^{+}$ T cells were costimulated either with wild-type HCT116 cells and either with CRISPR/Cas9 silenced EpCAM variants (KO clon2 and clon3) in the presence of CD3-EpCAM BsAb. Concentrations of IFNγ (**b**) (n=4 healthy donors) and IL-2 (**c**) (n=3 healthy donors) in the co-culture supernatants of the indicated conditions. Summary data are given as means ± s.e.m.

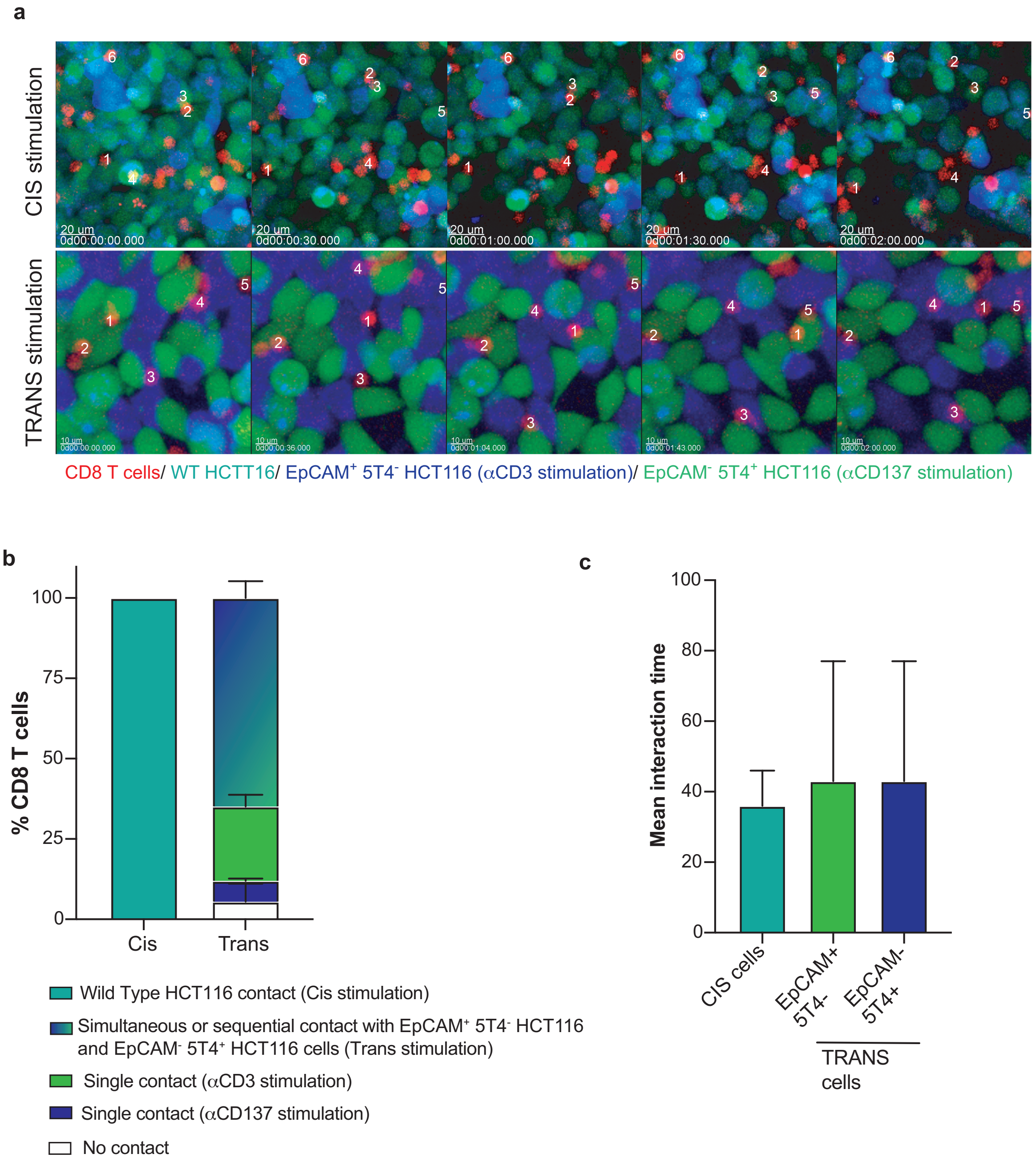


**Supplementary Figure 6. CD8$^+$ T cells interactions with tumor cells providing cis or trans costimulation. a**, representative frames from videos of CD8$^+$ T cells (RED) co-cultured with HCT116 5T4$^-$ EpCAM$^+$ (BLUE), 5T4$^+$ EpCAM$^-$ (GREEN) and WT (TURQUOISE) for 2 hours. Each digit in white indicates a single CD8$^+$ T-cell (scale 20 μm). **b**, quantification of CD8$^+$ T cell numbers that contacted with tumor cells in videos performed in a. CD8$^+$ T cells were followed over time and tracks were used to quantify the percentage of cells interacting with WT HCT116 (cis-stimulation), and percentage of cells interacting simultaneously or sequentially with 5T4$^-$ EpCAM$^+$ and 5T4$^+$ EpCAM$^-$ HCT116 (trans-stimulation) (scale 10 μm). **c**, summary of the T-cell:HCT116 contact duration during CD8$^+$ T cell stimulation with tumor cells as quantitively analysed in the time-lapse videos. Data are representative of two independent experiments. Data are given as mean ± s.e.m.

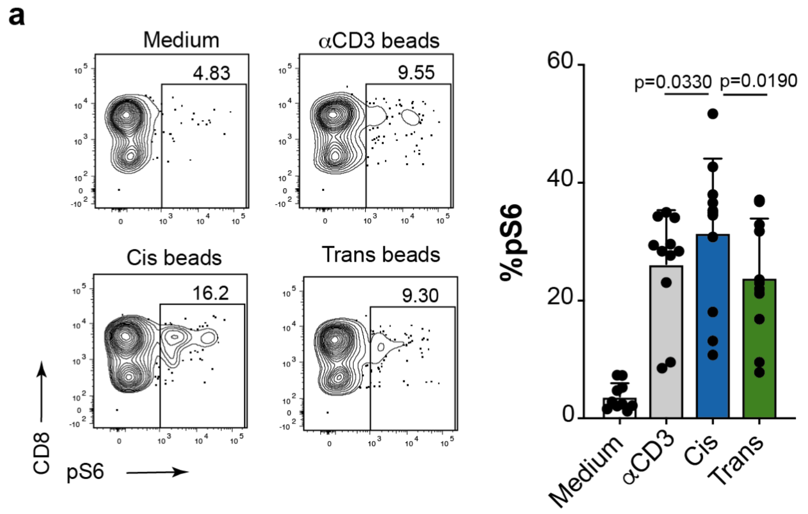


**Supplementary Figure 7. Cis CD137-costimulation is superior at inducing AKT-mTOR-S6 axis activation as compared to CD137-costimulation in trans. a,** representative flow cytometry dot plots showing pS6 phosphorylation in primary human CD8$^+$ T cells after CD137-costimulation with mAb coated beads (n=11 healthy donors). Summary data are given as mean ± s.e.m. Statistical significance was determined with one-way Anova with Dunn's multiple comparison test (one-sided).

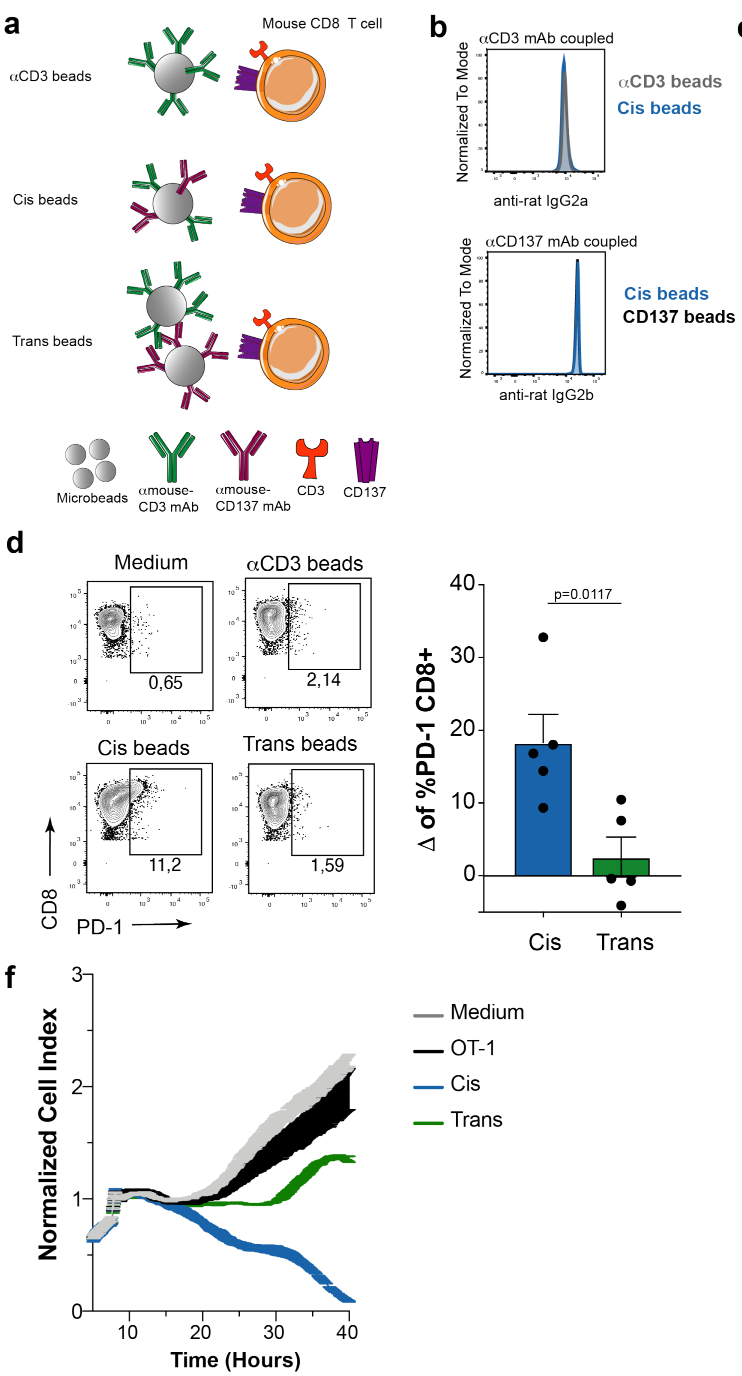


**Supplementary Figure 8. Cis CD137-costimulation superiority also in mouse CD8$^{+}$ T cells. a,** schematic representation of antibody-coated microbeads used to stimulate mouse CD8$^{+}$ T cells. **b**, semiquantitative FACS assessment of αCD3 and αCD137 mAbs coupled into the microbeads that were measured by anti-mouse IgG2a and anti-mouse IgG1 mAbs staining and detected by FACS. Representative histograms of the geometric mean fluorescence intensity for each condition. Mouse CD8$^{+}$ T cells isolated from spleens of naïve C57 mice were activated with mAb coated microbeads for 48 hours. Representative dot plots of cell-surface CD25 (**c**) and PD-1 (**d**) (n=5) and intranuclear Ki67 (**e**) (n=4) expression analyzed by flow cytometry at the indicated conditions. Cumulative data are shown as the difference between the value of cis and trans

conditions to which the anti-CD3 background was subtracted in each case. **f**, xCELLigence normalized cell index plot for B16.OVA target cells co-cultured with a 5:1 ratio (E:T) of OT-1 $CD8^+$ T cells pre-stimulated either with cis or trans mAb coated cells. Data have been normalized for the cell index value measured before addition of OT-1 effector cells. Data are given as mean ± s.e.m. Statistical significance was determined with paired t-test (two-sided).

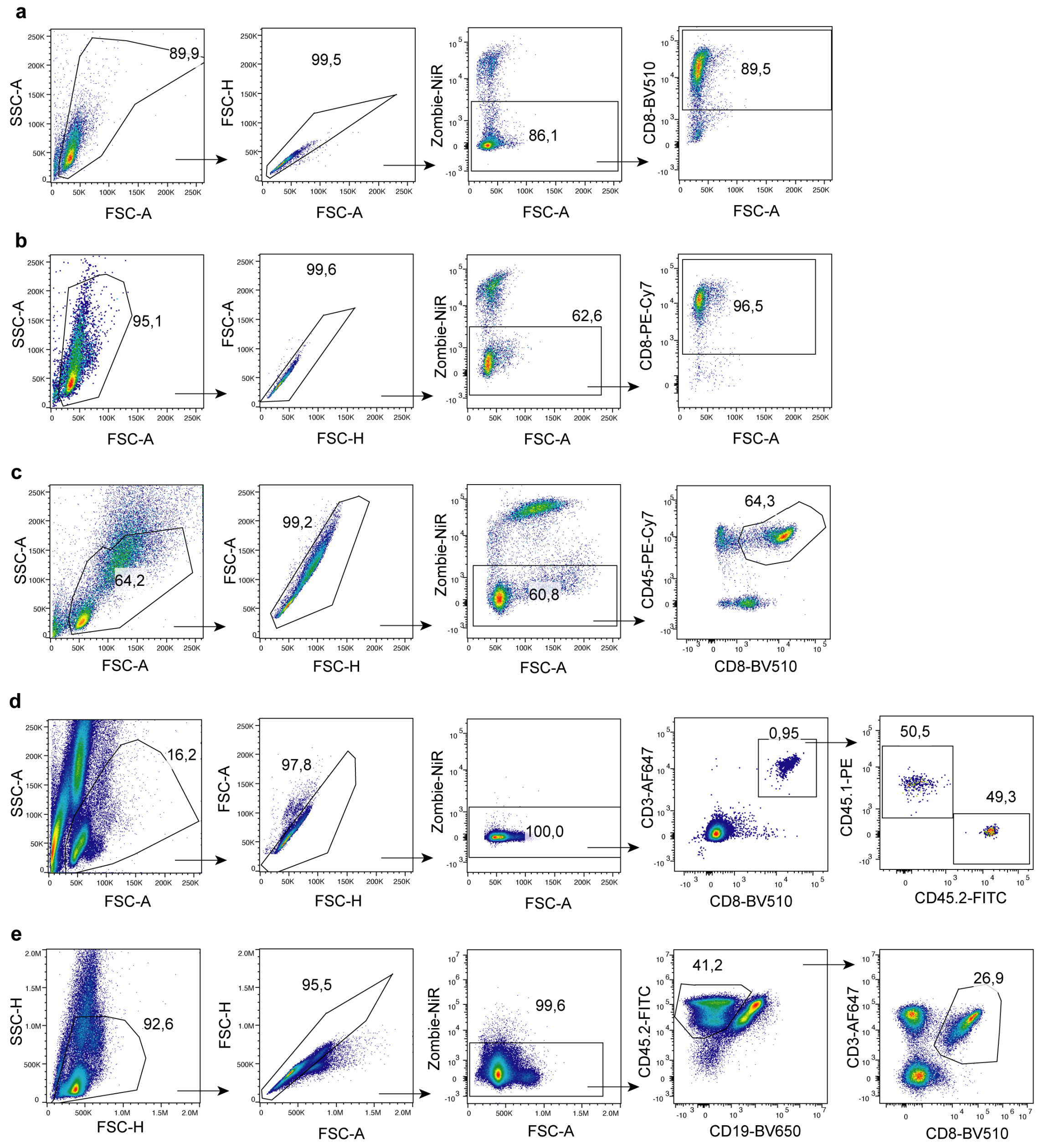


**Supplementary Figure 9. Gating strategies.** In all the cases, lymphocytes were gated in FSC/SCC plots, cells aggregations were excluded in an FSC-H/FSC-A plots and dead cells were discriminated based on a Live/Dead (Zombie-NiR) plot. Out of the live gate, $CD8^{+}$ T cells were gated with an anti-human (**a,** human PBMCs) or anti-mouse (**b,** mouse spleen) antibody. **c**, in the case of co-culture experiments of human $CD8^{+}$ T cells with HCT116 cell variants, $CD8^{+}$ T cells were gated on $CD8^{+}CD45^{+}$ expression. **d**, adoptively transferred mouse CD45.1/CD45.2 $CD8^{+}$ T cells into $Rag1^{-/-}$ mice, live cells were gated in a CD3/CD8 plot. Out of the double $CD3^{+}CD8^{+}$ positive population, CD45.1 and CD45.2 single positive population, were gated according to a CD45.1/CD45.2 plot. **e**, for the detection of OVA-specific $CD8^{+}$ T cells in the

blood of mice following immunization, $CD19^+$ cells were excluded from the viability gate, with a $CD45^+CD19^-$ gate, followed by a $CD3^+CD8^+$ gate.

**Supplementary Table 1. mAb coated microbeads for human T cells**

| Beads | Antibody coupled | Concentration |
|---|---|---|
| αCD3 beads | Anti-human CD3 | 2 μg |
| | Anti-mouse IgG1 | 8 μg |
| Cis beads | Anti-human CD3 | 2 μg |
| | Anti-human CD137 | 8 μg |
| αCD137 beads | Anti-mouse IgG2a | 2 μg |
| | Anti-human CD137 | 8 μg |

**Supplementary Table 2. CD137L coated microbeads for human T cells**

| Beads | Antibody coupled | Concentration |
|---|---|---|
| αCD3 beads | Anti-human CD3 | 4 μg |
| CD137L Cis beads | Anti-human CD3 | 4 μg |
| | CD137L | 6 μg |
| CD137L beads | Anti-mouse IgG2a | 4 μg |
| | CD137L | 6 μg |

**Supplementary Table 3. mAb coated microbeads for mouse T cells**

| Beads | Antibody coupled | Concentration |
|---|---|---|
| αCD3 beads | Anti-mouse CD3 | 2 μg |
| | Anti-rat IgG2a | 8 μg |
| Cis beads | Anti-mouse CD3 | 2 μg |
| | Anti-mouse CD137 | 8 μg |
| αCD137 beads | Anti-rat IgG2b | 2 μg |
| | Anti-mouse CD137 | 8 μg |

**Supplementary Table 4. Oligonucleotides used for gRNA**

| Gene target | Number | Sequence (5'-3´) |
|---|---|---|
| 5T4 | 1 | TAATACGACTCACTATAGCAGGTTGCGGAAGGACACGTGTTT<br>TAGAGCTAGAAATAGCAAGTTAAAATAAGGCTAGTCCGTTAT<br>CAACTTGAAAAAGTGGCACCGAGTCGGTGCTTTT |
| | 2 | TAATACGACTCACTATAGCGTTAACCGCAATCTGACCGGTTTT<br>AGAGCTAGAAATAGCAAGTTAAAATAAGGCTAGTCCGTTATC<br>AACTTGAAAAAGTGGCACCGAGTCGGTGCTTTT |
| Epcam | 1 | TAATACGACTCACTATAGGGCAAAAGTCGCCGTCGCCGGTTT<br>TAGAGCTAGAAATAGCAAGTTAAAATAAGGCTAGTCCGTTAT<br>CAACTTGAAAAAGTGGCACCGAGTCGGTGCTTTT |
| | 2 | TAATACGACTCACTATAGGATCCTGACTGCGATGAGAGGTTTT<br>AGAGCTAGAAATAGCAAGTTAAAATAAGGCTAGTCCGTTATC<br>AACTTGAAAAAGTGGCACCGAGTCGGTGCTTTT |

**Supplementary Table 5. Oligonucleotides used for T7E1 cleavage assay**

| Gene target | Number | Direction | Sequence (5'-3´) |
|---|---|---|---|
| 5T4 | 1 | Forward | GCAACCACTTCCTTTACCTGCC |
| | 2 | Reverse | AAGAATCGGGTCACAGTCCAGG |
| 5T4 | 1 | Forward | TCTCCTCGTCTTCTCCCACCTC |
| | 2 | Reverse | AGAAAGCGAAGGGACTGAGGTC |
| EpCAM | 1 | Forward | GGCCACAGAGTGAGACCCTATC |
| | 2 | Reverse | ACTCTTTCCAACTCAAGGCACAT |
| EpCAM | 1 | Forward | GCCAGTGTACTTCAGTTGGTGC |
| | 2 | Reverse | TCTGTTCTTCTGACCCCAGCAG |
