# Supplementary material for: CD137 (4-1BB) costimulation of CD8+ T cells is more potent when provided in cis than in trans with respect to CD3-TCR stimulation
Source: Nat Commun. 2021 Dec 15;12:7296. doi: 10.1038/s41467-021-27613-w (PMC8674279; doi:10.1038/s41467-021-27613-w)
Supplement: Supplementary file 3 — Description of Additional Supplementary Files [file 41467_2021_27613_MOESM3_ESM.pdf]

Description of Additional Supplementary Files

File Name: Supplementary Movie 1
Description: Related to FigS3. Time-lapse confocal microscopy videos showing CD8$^+$ T cells (red) interacting with mAb coated beads. Microbeads coated with �CD3-AF647 mAb are shown in blue, microbeads coated with �CD137-AF488 are shown in green, and microbeads coated with both �CD3-AF647 and �CD137-AF488 mAbs are shown in turquoise. CD8$^+$ T cells were followed over time for 8 hours and tracks were used to analyse the percentage of cells interacting with the different mAb coated microbeads. Circles indicate individual CD8$^+$ T cells interacting with mAb coated microbeads. Frames were obtained every 4 minutes.

File Name: Supplementary Movie 2
Description: Related to FigS5. Time-lapse confocal microscopy videos showing CD8$^+$ T cells (red) interacting with wild-type HCT116 cells (turquoise) or trans-costimulation with combinations of the alternatively silenced variants (5T4$^-$EpCAM$^+$, blue; 5T4$^+$EpCAM$^-$, green) in the presence of CD3-EpCAM and ALG.APV-527 BsAbs. CD8$^+$ T cells were followed over time for 2 hours and tracks were used to analyse the percentage of CD8$^+$ T cells interacting with the different cell variants. Frames were obtained every 3 minutes.
