# Supplementary material for: CD137 (4-1BB) costimulation of CD8+ T cells is more potent when provided in cis than in trans with respect to CD3-TCR stimulation
Source: Nat Commun. 2021 Dec 15;12:7296. doi: 10.1038/s41467-021-27613-w (PMC8674279; doi:10.1038/s41467-021-27613-w)
Supplement: Supplementary file 6 — Reporting Summary [file 41467_2021_27613_MOESM6_ESM.pdf]

nature research

Corresponding author(s): Ignacio Melero and Itziar Otano

Last updated by author(s): Oct 25 2021

Reporting Summary

Nature Research wishes to improve the reproducibility of the work that we publish. This form provides structure for consistency and transparency in reporting. For further information on Nature Research policies, see our Editorial Policies and the Editorial Policy Checklist.

Statistics

For all statistical analyses, confirm that the following items are present in the figure legend, table legend, main text, or Methods section.

n/a	Confirmed	
☐	☒	The exact sample size (n) for each experimental group/condition, given as a discrete number and unit of measurement
☐	☒	A statement on whether measurements were taken from distinct samples or whether the same sample was measured repeatedly
☐	☒	The statistical test(s) used AND whether they are one- or two-sided<br>*Only common tests should be described solely by name; describe more complex techniques in the Methods section.*
☐	☒	A description of all covariates tested
☐	☒	A description of any assumptions or corrections, such as tests of normality and adjustment for multiple comparisons
☐	☒	A full description of the statistical parameters including central tendency (e.g. means) or other basic estimates (e.g. regression coefficient) AND variation (e.g. standard deviation) or associated estimates of uncertainty (e.g. confidence intervals)
☐	☒	For null hypothesis testing, the test statistic (e.g. F, t, r) with confidence intervals, effect sizes, degrees of freedom and P value noted<br>*Give P values as exact values whenever suitable.*
☒	☐	For Bayesian analysis, information on the choice of priors and Markov chain Monte Carlo settings
☒	☐	For hierarchical and complex designs, identification of the appropriate level for tests and full reporting of outcomes
☒	☐	Estimates of effect sizes (e.g. Cohen's d, Pearson's r), indicating how they were calculated

Our web collection on statistics for biologists contains articles on many of the points above.

Software and code

Policy information about availability of computer code

Data collection	Flow cytometry data were collected by BD FACSDiva v9.0, CytoflexS or CytoflexLX. Confocal images and time-lapse videos were taken in a LSM800 microscopy. In vivo Bioluminiscence images were acquired with PhotonIMAGER. In vitro bioluminiscence signal was acquired in an Orion L Microplate<br>Luminometer.
Data analysis	Flow cytometry data were analyzed with FlowJoTM v.10.6.2 software for Mac. GraphPad Prism version 8.2.1 for Mac. Confocal images were analyzed with ImageJ FIJI v1.51s. Western densitometry was performed using Image Studio Lite v5.2.5. Microarray data was normalized with R/Bioconductor v3.5.1, affy R package: affy_1.60.0, Limma R package: limma_3.38.3 and analyzed with Ingenuity v52912811. Confocal timelapse videos were analyzed with Imaris software v9.8 (Bitplane). The single guide RNAs (sgRNAs) were designed with the online Benchling. software. The MoFlo Astrios EQ Cell Sorter's Summit v62 software was used to collect the data of the single-cell sorting experiments.

For manuscripts utilizing custom algorithms or software that are central to the research but not yet described in published literature, software must be made available to editors and reviewers. We strongly encourage code deposition in a community repository (e.g. GitHub). See the Nature Research guidelines for submitting code & software for further information.

Data

Policy information about availability of data

All manuscripts must include a data availability statement. This statement should provide the following information, where applicable:

- Accession codes, unique identifiers, or web links for publicly available datasets
- A list of figures that have associated raw data
- A description of any restrictions on data availability

The authors declare that the data supporting the findings of this study are available within the paper and its supplementary information files or available from the

authors upon reasonable request. Microarray data are available in the National Center for Biotechnology Information Gene Expression Omnibus (NCBI-GEO) under accession number GSE158041. https://www.ncbi.nlm.nih.gov/geo/query/acc.cgi?acc=GSE158041. The remaining data supporting the findings of this study are available in the Source Data file.

Field-specific reporting

Please select the one below that is the best fit for your research. If you are not sure, read the appropriate sections before making your selection.

☒ Life sciences ☐ Behavioural & social sciences ☐ Ecological, evolutionary & environmental sciences

For a reference copy of the document with all sections, see nature.com/documents/nr-reporting-summary-flat.pdf

Life sciences study design

All studies must disclose on these points even when the disclosure is negative.

Sample size	Sample size was calculated with the the G Power software (http://www.gpower.hhu.de/).
Data exclusions	No data were excluded from the analyses.
Replication	All experiments have been repeated at least twice with similar results except the experiment with RAG1-/- mice in figure 4 and the microarray in figure 5. Experiments in vitro were performed at least with duplicates.
Randomization	Animals were randomly assigned to the groups. Human PBMCs were obtained from random healthy donors independently of sex, age and any other characteristics and were randomly allocated into experimental groups.
Blinding	This manuscript is focused on preclinical research. Assays were not blinded because the administration, sample collection and processing were carried out by the same researchers.

Reporting for specific materials, systems and methods

We require information from authors about some types of materials, experimental systems and methods used in many studies. Here, indicate whether each material, system or method listed is relevant to your study. If you are not sure if a list item applies to your research, read the appropriate section before selecting a response.

Materials & experimental systems

n/a	Involved in the study	
☐	☒	Antibodies
☐	☒	Eukaryotic cell lines
☒	☐	Palaeontology and archaeology
☐	☒	Animals and other organisms
☐	☒	Human research participants
☒	☐	Clinical data
☐	☒	Dual use research of concern

Methods

n/a	Involved in the study	
☒	☐	ChIP-seq
☐	☒	Flow cytometry
☒	☐	MRI-based neuroimaging

Antibodies

Antibodies used

Human:
Flow cytometry: anti-CD8-BV510 (SK1, 344732, Biolegend), anti-CD25-APC (BC96, 302610, Biolegend), anti-CD45-PE-Cy7 (HI30, 304016, Biolegend), anti-PD1-PerCPCy5.5 (EH12.2H7, 329914, Biolegend), Ki67-AF488 (Ki-67, 350508, Biolegend), T-bet-BV421 (eBio4B10, 644816, Biolegend), anti-EpCAM-PerCPCy5.5 (9C4, 324214, Biolegend), gH2AX-PE (2F3, 613412, Biolegend), gH2AX-AF647 (2F3, 560447, BD Bioscience), pS6-AF647 (D57.2.2E, 4851, Cell Signaling) , anti-Bcl-xL-PE (7B2.5, Southern Biotech), anti-5T4 (Alligator Bioscience).

Anti-CD3 mAb (OKT3, BE0001-2, Bio X cell) and anti-CD28 mAb (37.51, 102115, Biolegend) were used to stimulate CD8 T cells in vitro.

Western Blot: anti-IKBa (rabbit polyclonal; ab32518, Abcam), anti-mouse p65 (mouse monoclonoal, in house), anti-human p52/p100 (rabbit polyclonal, 4882S, Cell signaling), anti-human B-actin (rabbit polyclonal, A2066, Sigma) and anti-human C23 (MS-3, sc-8031, Santa Cruz).

Antibodies used to coat microbeads with human mAb: anti-human CD3 (OKT3, produced in house), anti-human CD137 (6B4, produced in house), anti-human CD3 (OKT3, BE0001-2, Bio X cell), mouse IgG2a (MOPC-173, 400202, Biolegend), mouse IgG1 (MOPC-21, 400102, Biolegend), anti-CD7 mAb (4H9/CD7,395602, Biolegend).

Mouse:
Flow cytometry: anti-CD3-BUV496 (145-2C11, 564661, BD Bioscience), anti-CD3-AF647 (17A2, 100209, Biolegend), anti-CD8-BV510

(53-6.7, 100751, Biolegend), anti-CD25-FITC (PC61, 102006, Biolegend), anti-CD25-APC (PC61, 102012, Biolegend), anti-CD45.1-PE (A20, 110708, Biolegend), anti-CD45.2-Pacific Blue (104, 109820, Biolegend), CD45.2-FITC (1:300, 109806), anti-PD1-PerCPCy5.5 (29F.1A12, 124334, Biolegend), Ki67-AF700 (16A8, 652420, Biolegend), T-bet-PE-Cy7 (eBio4B10, 25-5825-82, eBioscience), Eomes-eFluor450 (Dan11mag, 48-4875-82, eBioscience), anti-mouse CD16/32 (93, True stain FcX, 101320 Biolegend), anti-CD19-BV650 (115541, Biolegend) and iTAg Tetramer/PE-H-2 Kb OVA (SIINFEKL) (6D5, TB-5001-1, MBL).

Antibodies used to coat microbeads with mouse mAb: anti-mouse CD3 (17A.2, 100238, Biolegend), anti-mouse CD137 (BE0239, Bio X cell), anti-mouse CD137 (3H3, produced in house), rat IgG2a (RTK2758, 400533, Biolegend), rat IgG2b (RTK4530, 400637, Biolegend).

Other antibodies: anti-mouse IgG1-PE (RMG1-1, 406608, Biolegend), anti-mouse IgG2a-AF647 (Polyclonal, A21241, INVITROGEN), anti-rat IgG2a-AF647 (2A8F4, ab172333, abcam), anti-rat IgG2b-AF488 (2B 10A8, ab172334, abcam), donkey anti-rabbit AF647 IgG(H+L) (1964354, Invitrogen), anti-rabbit IgG HRP (170-6515, BioRad), goat anti-mouse IgG HRP (A0168, Sigma).

Validation

All commercial antibodies were used (assay and species) according to the manufacturer's instructions. Validation of flow cytometry antibodies was performed by the titration to determine the optimal concentration through the series of the dilutions: 1/800, 1/400, 1/200, 1/100, 1/50, 1/25. Optimal concentration was defined by calculating the stain index.

Eukaryotic cell lines

Policy information about cell lines

Cell line source(s)

The HCT116 cell line was obtained from ATCC. The CD137-Jurkat reporter cell line was included in the 4-1BB Bioassay (JA2351, Promega). B16-OVA cells were provided by Dr. Lieping Chen (Yale University, New Haven, CT) in November 2001.

Authentication

The B16.OVA cell line was authenticated by Idexx Radil (Case 6592-2012) in February 2012. We did not perform further authentication. However, the cell lines behaved as expected in vitro and in vivo and this behavior did not change.

Mycoplasma contamination

All cell lines were tested negative for mycoplasma contamination.

Commonly misidentified lines
(See ICLAC register)

We did not used commonly misidentified lines.

Animals and other organisms

Policy information about studies involving animals; ARRIVE guidelines recommended for reporting animal research

Laboratory animals

C57BL/6 mice (6-8 weeks old, male) were obtained from Envigo (Huntingdon, Cambridgeshire, UK) and maintained in the animal facility of Cima-Universidad de Navarra. Wild type C57 BL/6 CD45.1+ (6-8 weeks old, male), B6.129S7-Rag1tm1Mom/J (Rag1) (6-8 weeks old, male) and C;129S4-Rag2tm1.1Flv Il2rgtm1.1Flv/J (Rag2-/-IL2Rgc-/-) mice (6-8 weeks old, female) were bred at CIMA Universidad de Navarra in specific pathogen-free conditions. OT-1 and OT-1 CD45.1 (6-8 weeks old, male) were bred at CIMA Universidad de Navarra in specific pathogen-free conditions. Lights were on a 12h ON/OFF cycle, room temperature was set to 22°C with the variance of +/- 2°C and ambient humidity conditions.

Wild animals

The study did not involve wild animals.

Field-collected samples

The study did not involve samples collected from the field.

Ethics oversight

Experiments involving mice were approved by the Ethics Committee of the University of Navarra (R-030-19GN)

Note that full information on the approval of the study protocol must also be provided in the manuscript.

Human research participants

Policy information about studies involving human research participants

Population characteristics

PBMCs were obtained from 50 healthy white donors (21-50 years, males and females). All samples were obtained after informed consent from the healthy donors and Institutional Review Board approval and we have complied all ethical regulations.

Recruitment

Healthy donors were recruited by the Biobank. We randomly selected samples from healthy donors. All the samples were anonymized. potential bias of the recruitment is the underrepresentation of certain ethnic groups due to the demographics in Pamplona, Spain. No other self-selection biases are present.

Ethics oversight

This study was approved by the Regional Ethics Committee of the University of Navarra (2019-039) and Navarra Blood and Tissue Bank Navarrabiomed Biobank.

Note that full information on the approval of the study protocol must also be provided in the manuscript.

Dual use research of concern

Policy information about dual use research of concern

Hazards

Could the accidental, deliberate or reckless misuse of agents or technologies generated in the work, or the application of information presented in the manuscript, pose a threat to:

No	Yes	
☐	☐	Public health
☐	☐	National security
☐	☐	Crops and/or livestock
☐	☐	Ecosystems
☐	☐	Any other significant area

Experiments of concern

Does the work involve any of these experiments of concern:

No	Yes	
☐	☐	Demonstrate how to render a vaccine ineffective
☐	☐	Confer resistance to therapeutically useful antibiotics or antiviral agents
☐	☐	Enhance the virulence of a pathogen or render a nonpathogen virulent
☐	☐	Increase transmissibility of a pathogen
☐	☐	Alter the host range of a pathogen
☐	☐	Enable evasion of diagnostic/detection modalities
☐	☐	Enable the weaponization of a biological agent or toxin
☐	☐	Any other potentially harmful combination of experiments and agents

Flow Cytometry

Plots

Confirm that:

☒ The axis labels state the marker and fluorochrome used (e.g. CD4-FITC).

☒ The axis scales are clearly visible. Include numbers along axes only for bottom left plot of group (a 'group' is an analysis of identical markers).

☒ All plots are contour plots with outliers or pseudocolor plots.

☒ A numerical value for number of cells or percentage (with statistics) is provided.

Methodology

Sample preparation	Mouse spleens, bone marrows and lymph nodes were mechanically dissociated. Liver samples were incubated with 400 MandL/ml of collagenase and 50ug/ml of DNase (Roche) for 30 minutes at 37°C. Single-cell suspensions were prepared by mechanically disrupting the organs through a 70-µm cell strainer (Falcon).Liver cells were isolated by centrifugation with 35% Percoll (GE Healthcare). Cells were then subjected to red blood cell lysis (ACK Lysing Buffer, A1049201, ThermoFisher).<br>Blood from mice was collected in in 1.5ml tubes containing Heparin. Peripheral blood leukocytes were prepared by lysing erythrocytes with the red blood BD FACS™ lysing solution.<br>Human PBMCs from healthy donors were enriched using Ficoll-Paque Plus (GE Healtcare, menlo Park, CA) and isolated by density gradient centrifugation.
Instrument	FACSCanto II (BD), CytoflexS, Cytoflex XL (Beckman Coulter) and MoFLo (Astrios, Beckman Coulter)
Software	FlowJo 10.6.2 (Tree Star, Ashland, OR), CytExpert and Summit v62 (Beckman Coulter)
Cell population abundance	HCT116 cells electroporated with Cas9/RNPs targeting EpCAM or 5T4 were single-sorted and cloned. The single-cell clones were evaluated 3 days after sorting to exclude multiple cell contamination. Cells were cultured until confluence. For flow cytometry analysis, cells were harvested with AccutaseTM (Gibco) and stained for EpCAM and 5T4 expression. HCT116 silenced variants were selected when surface EpCAM or 5T4 expression was not detected. Wild type HCT116 cell line was used as a positive control for EpCAM and 5T4 expression.<br>For microarray analysis, high-purity (>95%) CD8 T was required.
Gating strategy	CD8 T cells were magnetically sorted by negative selection either from human PBMCs or from the spleens of naïve mice. In all cases, lymphocytes were gated in FSC/SSC plots, cell aggregations were excluded in FSC-H/FSC-A plots and dead cells were discriminated based on a Live/Dead (Zombie NiR, BioLegend or PROMOFLUOR-840, Fisher) plot. Out of the live gate, CD8 T cells were gated with an anti-human or anti-mouse CD8 antibody.<br><br>In the case of co-cultures experiments of hCD8s with HCT116 cell variants, CD8 T cells were gated based on CD8 and CD45 expression.,

In order to discriminate adoptively transfered mouse CD45.1/CD45.2 CD8 T cells into Rag1-/- recipients, live cells were gated in a CD3/CD8 plot. Out of the double CD3+CD8+ positive population, CD45.1 or CD45.2 single population were gated using a CD45.1/CD45.2 plot.

For the detection of OVA-specific CD8 T cells in the blood of mice following immunization, a preliminary FSC/SSC gate was utilized to gate on the morphology of lymphocytes. Subsequently a singlets gate (FSC-H vs FSC-A) was used to exclude the doublets followed by the viability gate (Live/Dead UV vs FSC-A) to exclude dead cells. From this population, CD19+ cells were excluded with a CD45+CD19- gate, followed by a CD3+CD8+ gate.

☒ Tick this box to confirm that a figure exemplifying the gating strategy is provided in the Supplementary Information.
